# Using the Smith-Watson-Topper Parameter and Its Modifications to Calculate the Fatigue Life of Metals: The State-of-the-Art

**DOI:** 10.3390/ma15103481

**Published:** 2022-05-12

**Authors:** Tadeusz Łagoda, Sabrina Vantadori, Karolina Głowacka, Marta Kurek, Krzysztof Kluger

**Affiliations:** 1 Faculty of Mechanical Engineering, Opole University of Technology, 45-758 Opole, Poland; k.glowacka@po.edu.pl (K.G.); ma.kurek@po.edu.pl (M.K.); k.kluger@po.edu.pl (K.K.); 2 Department of Engineering & Architecture, University of Parma, 43124 Parma, Italy; sabrina.vantadori@unipr.it

**Keywords:** stress, strain, energy parameter, critical plane, fatigue

## Abstract

The Smith-Watson-Topper parameter (SWT) in its original form was designed to estimate the fatigue life of metal materials in a uniaxial load state (tension–compression) in the range up to fatigue crack initiation, with non-zero mean values. This parameter is based on the analysis of both stress and strain. Therefore, the stress–strain criterion is the focus, rather than the energy criterion. This paper presents the original SWT model and its numerous modifications. The first part presents different versions of this parameter defined by the normal parameters. Then, it presents versions defined through the tangent parameter and the most promising parameter defined through the tangent and normal parameters. It was noted that the final form of the equivalent value is defined either by stress or by an energy parameter. Therefore, the possible characteristics from which the fatigue life can be determined are also presented.

## 1. Introduction

The problem of fatigue life assessment has been worked on for many years, but has not been solved so far. A good understanding of fatigue processes is particularly important in such industries where a possible failure is a significant threat to human health and life. This is especially true in the power and transport industries. One of the important issues that has not been solved is the reduction in the complex stress state to its uniaxial equivalent. For this purpose, various types of strength hypotheses which result in correlations for the equivalent curve expressed in stress or strain have been proposed. As a result, amplitudes of stresses or strains are obtained, and on this basis, fatigue life can be determined. The criteria of multiaxial fatigue originating in the abovementioned hypotheses can be divided according to the physical nature of the failure parameter into stress criteria, strain criteria, and energy (stress–strain) criteria. Many stress criteria have been developed for this purpose. These criteria are dedicated to a large number of cycles to failure, i.e., when plastic deformations take on very small values and can be omitted from the computational process. In the case of large plastic deformations, strain criteria that reduce the complex state of strain to the uniaxial equivalent have been proposed.

It can be seen that the proposed SWT parameter and its modifications are very up to date in both global and national science. The topicality of the problem was noticeable in the last webinar that took place on the Zoom platform on 7 May 2021. The title of the seminar was “Discussion on the nature, limitations and successes of the SWT fatigue damage parameter”, and it was presented by Prof. Tim Topper and Prof. Grzegorz Glinka from the University of Waterloo, Canada. Here, it should be noted that at the meeting, only the problem of uniaxial fatigue was discussed. On the other hand, this paper aims to verify the model for a complex load condition. For this reason, the problem is very important for the development of science, especially in the field of mechanical engineering.

So-called energy criteria have been developed recently. The first models refer to the Garuda proposal [1], which calculates the cumulative plastic energy as:

(1) $$\Delta W_{p}=\overset{}{\underset{cycle}{\int }}\sigma_{ij}d\varepsilon_{ij}^{p}$$

where $\sigma_{ij}$ is stress tensor and $d\varepsilon_{ij}^{p}$ is plastic strain tensor.

This approach is based upon the idea of relating fatigue life to the plastic work during a cycle of the loading. The simple addition of plastic energy, e.g., from tension–compression and torsion, insufficiently describes the fatigue process. Corrective coefficients had to be introduced for the shear strain energy (usually with correction factor *ω* = 0.5). Other proposals concern the addition of elastic or hydrostatic energy:

(2) $$\Delta W=\Delta W_{p}^{}+\Delta W_{e}^{}+\Delta W_{h}^{}$$

including hydrostatic energy based on hydrostatic stress:

(3) $$\Delta W_{h}^{}=\frac{3\left(1-2\vartheta \right)}{2E}\sigma_{h}^{2}$$

where *E* is Young’s modulus of elasticity, *σ_h_* is hydrostatic stress, and *υ* is the elastic Poisson’s ratio.

However, it seems that the past belongs to the Smith-Watson-Topper (SWT) parameter concept [2], which is still being developed. In this case, the stress–strain criterion is the focus, rather than the energy criterion, because the calculated parameter is not energy. It should be noted that regardless of whether it is the value of energy or the stress–strain parameter, the unit is MPa. According to [2], the stress–strain function governing fatigue is extended to include the effect of mean stress. In this paper, a procedure for cumulative damage summation in terms of this function is presented. The proposal was tested against various metals (steels and aluminum alloys) and loading conditions. The details of this parameter will be described in the next section. Then, various modifications will be presented. The third section will describe the modifications in the plane defined by the normal stress values. The fourth section provides an overview of the damage parameters, including the modifications in the plane defined by the shear stress values. The SWT parameter defined by the sum of the normal and shear stress values is presented in Section 5.

The verification of various models can be found in many works, especially in review papers [3,4,5,6,7,8,9,10,11,12,13,14,15,16,17,18]. There are also models dedicated to anisotropic materials such as composites, but we limited the review to metals. The next section presents the fatigue characteristics used to determine the fatigue life. Section 2, Section 3, Section 4, Section 5 and Section 6 present the method of damage parameter (*DP*) determination depending on the selected model of fatigue life determination. The detailed description of the analyzed models will be presented in Section 7. The damage parameter can be defined by determining the equivalent stress or energy parameter. In both cases, these parameters can be defined by different physical magnitudes (shear and/or normal stress or by the tangential and/or normal component).

The aim of this work was to review the models used so far, which are based only on the original Smith-Watson-Topper parameter proposal. All these models are defined in the critical plane that is defined differently, depending on the model.

## 2. Definition of Original Smith-Watson-Topper Parameter

The original Smith-Watson-Topper parameter [2] can be noted as damage parameter, *DP_n_*:

(4) $$\;DP_{n}=\sigma_{max}\varepsilon_{a}$$

where $\sigma_{max}$ is maximum normal stress, and $\varepsilon_{a}$ is normal strain amplitude, or in a modified version as the equivalent stress value:

(5) $$DP_{\sigma }={\left(\sigma_{max}\varepsilon_{a}E\right)}^{1/2}={\left(\sigma_{max}\sigma_{a}\right)}^{1/2}$$

where

(6) $$\sigma_{max}=\sigma_{a}+\sigma_{m}$$

*E* is Young’s modulus.

Parameters in version (4) or (5) have been successfully used to describe uniaxial cyclic fatigue with a non-zero mean value over the full range of fatigue life. Here, we can mention six monographs [19,20,21,22,23,24], which present the results of cyclic tests for numerous materials using the SWT parameter, where one function is used to describe fatigue tests with zero and non-zero average values.

**Figure 1:**
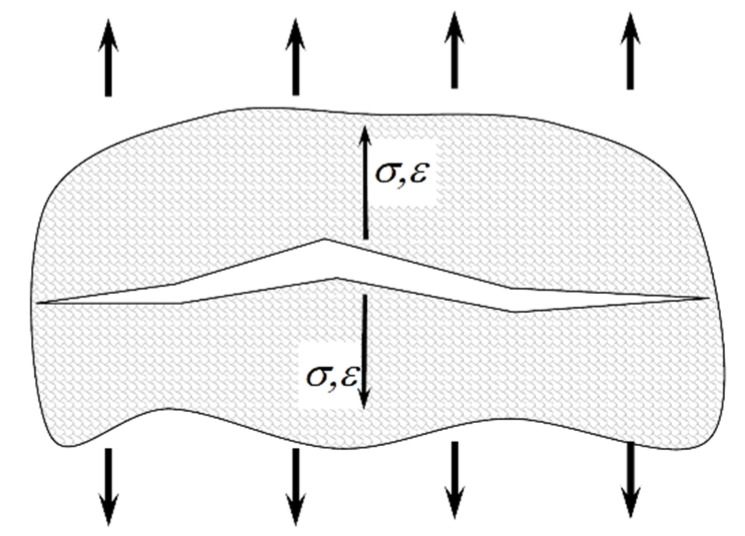
Interpretation of the SWT parameter defined by the normal component.

This parameter was later modified many times and used to describe fatigue life in a complex load condition. The interpretation of the SWT parameter defined by the normal component is shown in Figure 1.

## 3. The Smith-Watson-Topper Parameter Defined by Normal Values

A wider analysis of the Smith-Watson-Topper parameter (4) related to the influence of the mean value can be found in [25], where Zhang and Akid analyzed the SWT parameter based not only on shearing or tension–compression, but also on tension–compression with static torsion and cyclic torsion with static tension. These non-standard fatigue tests allowed the authors to effectively verify the proposed model. In [26], the authors evaluated the mean strain influence on the total number of cycles to fatigue failure of AA7175-T1 aluminum alloy, based on empirical relationships developed by SWT, by Morrow and Walker, which were used to adjust the experimental data and predict the fatigue life behavior at different mean strain values applied during cyclic loading. The impact of the mean value modified in relation to the proposal (5) can be written, according to Walker’s proposal [27], as:

(7) $$DP_{\sigma }=\sigma_{max}^{1-\gamma }\sigma_{a}^{\gamma },$$

or by Lv et al. [28] in the form of:

(8) $$DP_{n}=2\gamma \sigma_{max}\varepsilon_{a}{}^{},$$

where

(9) $$\gamma =0.5\pm \frac{\sigma_{y}-\sigma_{u}}{\sigma_{y}+\sigma_{u}},$$

where γ is the material-dependent exponent, *σ_y_* is the yield strength, and *σ_u_* is the ultimate strength.

In this research, considering the high accuracy of mean stress correction and the difficulty in obtaining the material parameter of the Walker method, a practical method is proposed to describe the material parameter of this method. According to [28], the proposed strain-life model provides more accurate life prediction results than the SWT parameter method.

In [29], the results of fatigue tests of SAE 1045 steel with different mean values were analyzed. Different models were used for this purpose, including two original SWT parameters from (5) and two modifications of this parameter proposed by Bergmann et al. [30]:

(10) $$DP_{\sigma }={\left((\sigma_{a}+k\sigma_{m})\varepsilon_{a}E\right)}^{1/2}$$

This modification is based on the fact that the value of the *k*-factor does not have to be 1 according to Equation (6).

In [30], the authors proposed that *k* = 0.45 when analyzing the results obtained for carbon steel, and [31] showed that better compliance with the experiment was achieved for *k* = 0.4. In [29], the authors showed that, for 1045 steel, the smallest error in relation to the experiment was obtained for *k* = 1.2. According to Roberts and Erdogan [32]:

(11) $$DP_{\sigma }={\left({\left(\sigma_{a}\right)}^{\gamma }\;{\left(\sigma_{max}\right)}^{1-\gamma }\varepsilon_{a}E\right)}^{1/2}.$$

In [31], the authors proposed using an exponent for *γ* = 0.5. Socie [33] modified the original form of the SWT parameter in the plane of maximum strain range:

(12) $$DP_{\sigma }={\left\{{\left(\sigma_{max}\frac{\Delta \varepsilon_{max}}{2}E\right)}^{1/2}\right\}}_{},$$

where the maximum value of the stress is determined in the plane in which we are dealing with the largest range of strain. In this paper, the failure mode is shown to be dependent on material, strain range, and hydrostatic stress state. Tests to support these models were conducted with Inconel 718, SAE 1045, and AISI Type 304 stainless-steel tubular specimens in strain control. Navarro et al. [34] used different models, including the SWT-based model in its classic form, for analysis based on fatigue fretting tests. Similar assumptions can be found in [7], where the original version was also modified:

(13) $$DP_{\sigma }={\left\{{\left(\sigma_{max}\varepsilon_{a}E\right)}^{1/2}\right\}}_{max},$$

and a plane was sought in which the product of the maximum stress and the strain amplitude reaches the maximum value. In addition, Szolwinski and Farris [35] used the SWT parameter to analyze fatigue fretting tests, where the maximum stress is defined by the conditions under which the tests were performed, and the strain range was determined by assuming a flat strain state.

## 4. Smith-Watson-Topper Parameter Defined by Shear Values

Yu et al. [36] formed a proposal in relation to the original form of SWT (5) expressed for strain and normal stress. In this model, the decisive factor for failure is the product of the maximum shear strains and the amplitude of these strains:

(14) $$DP_{s}={\left(\gamma_{max}\gamma_{a}G\right)}^{1/2}.$$

where $\gamma_{max}$ is maximum shear strain, $\gamma_{a}$ is shear strain amplitude, and *G* is shear modulus.

Suman et al. [37] proposed another form of the failure (damage) parameter as:

(15) $$DP_{\tau }={\left(G\Delta \gamma \right)}^{w}\tau_{max}^{1-w}\left[1+k\frac{{\left(\sigma \tau \right)}_{max}}{\sigma_{o}^{2}}\right].$$

where $\tau_{max}$ is maximum shear stress.

**Figure 2:**
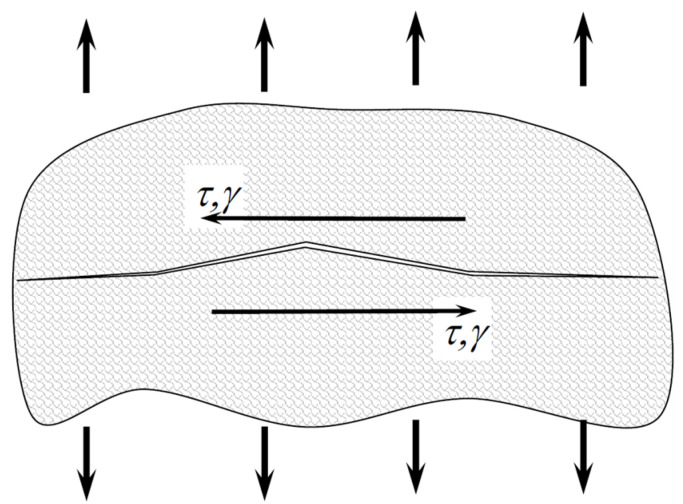
Interpretation of the SWT parameter defined by the shear component.

The interpretation of the SWT parameter defined by the shear component is shown in Figure 2.

Since Equation (15) is defined in the amplitude of the shear strain, the equation can be written in a modified form so that it is compatible with other equations, in the form of:

(16) $$DP_{\tau }={\left(\frac{G\Delta \gamma }{2}\right)}^{w}\tau_{max}^{1-w}\left[1+k\frac{{\left(\sigma \tau \right)}_{max}}{\sigma_{o}^{2}}\right]$$

where *G* is shear modulus, ∆*γ* is shear strain range, *τ_max_* is maximum shear stress, (*στ*)*_max_* is the maximum shear and tensile stress product value, *σ_o_* is a factor used to maintain unit consistency, and *w* and *k* are material fitting parameters.

From the analysis of the results presented in [37], it can be concluded that there is a very good compliance with the results of experimental studies.

Glinka et al. [38] proposed their damage parameter in the form of:

(17) $$DP_{s}=\frac{\Delta \gamma_{12}}{2}\frac{\Delta \sigma_{12}}{2}{\left(\frac{{\tau^{\prime }}_{f}}{{\tau^{\prime }}_{f}-\sigma_{12}^{max}}+\frac{{\sigma^{\prime }}_{f}}{{\sigma^{\prime }}_{f}-\sigma_{22}^{max}}\right)}_{max}$$

Kanchanomai et al. [39] proposed a modified Glinka’s criterion in the form:

(18) $$DP_{s}=\frac{\Delta \gamma_{12}}{2}\frac{\Delta \sigma_{12}}{2}\left(\frac{1}{1-\frac{\sigma_{12}^{max}}{{\tau^{\prime }}_{f}}}+\frac{1}{1-\frac{\sigma_{22}^{max}}{{\sigma^{\prime }}_{f}}}\right)$$

In this paper, the fatigue mechanisms were studied by scanning electron microscopy (SEM) examination of the polished surface of specimens and fracture surfaces. Wedge cracking due to grain boundary sliding was the dominant mechanism in the low strain rate regime, while extensive cavitation was observed on the colony boundaries at a high strain rate regime.

Hao et al. presented another proposal in [40], which has the form of:

(19) $$DP_{s}=\frac{\Delta \gamma_{eq}}{2}\frac{\Delta {\tau^{\prime }}_{eq}}{2}$$

where,

(20) $$\frac{\Delta \gamma_{eq}}{2}=\sqrt{{\left(\frac{\Delta \gamma_{ns}}{2}\right)}^{2}+3{\left(\frac{\Delta \varepsilon_{n}}{2}\right)}^{2}}$$

(21) $$\frac{\Delta {\tau^{\prime }}_{eq}}{2}=\sqrt{\alpha {\left(\frac{\Delta \tau_{ns}}{2}\right)}^{2}+\beta {\left(\frac{\Delta \sigma_{n}}{2}\right)}^{2}}$$

(22) $$\alpha =1-\frac{1}{3}\vartheta \frac{\sigma_{a,max}}{{\sigma^{\prime }}_{f}}$$

(23) $$\beta =\frac{1}{3}\vartheta \frac{\sigma_{a,max}}{{\sigma^{\prime }}_{f}}$$

where *σ_a,max_* is maximum normal stress in the maximum shear strain plane.

Based on the critical plane approach, this article develops a new damage parameter through combing the equivalent strain energy aspect for multiaxial fatigue analysis, which includes no additional fitted parameters and overcomes the deficiency of using only equivalent stress/strain criterion separately under multiaxial loadings.

## 5. The Smith-Watson-Topper Parameter Defined by Normal and Shear Values

Chen [41] proposed adding a tangential part to the original model, namely the product of the shear strain range and the shear stress range. He described his proposals in two ways.

The first way concerns the summation in the plane of the maximum range of normal strain:

(24) $$DP_{ns}=\Delta \varepsilon_{1}^{max}\Delta \sigma_{1}+\Delta \gamma_{1}^{}\Delta \tau_{1},$$

and the other way concerns the summation in the plane of maximum shear strain:

(25) $$DP_{ns}=\Delta \varepsilon_{n}^{}\Delta \sigma_{n}+\Delta \gamma_{}^{max}\Delta \tau_{1}.$$

**Figure 3:**
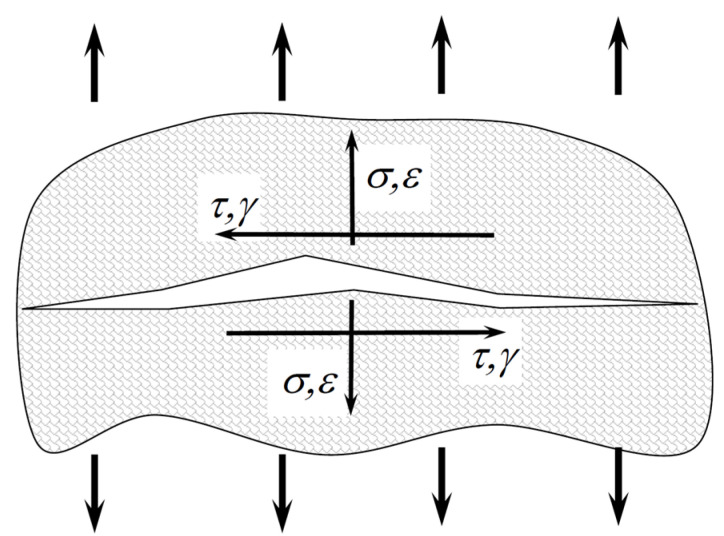
Interpretation of the SWT parameter defined by the normal and shear components.

The interpretation of the SWT parameter defined by the normal and shear components is shown in Figure 3.

Gupta et al. [42] (quotation from [11]) proposed modifying Chen’s proposal [41] in the critical plane determined by the range of maximum normal stresses, as:

(26) $$DP_{ns}=\Delta \varepsilon_{1}^{}\Delta \sigma_{1}^{max}+\Delta \gamma_{1}^{}\Delta \tau_{1}.$$

The predicted fatigue life using the developed parameter, for varying principal direction tension–torsion tests, was found to be in good agreement and mostly on the conservative side.

Similar to Chen [41], Calvo et al. [43] proposed (based on the concept of virtual strain Energy by Liu et al. [44]) modifying Equations (24) and (25) by taking into account the influence of non-zero mean values and defining the critical plane in a different way.

The first Calvo proposal [43] is defined in the plane of the product of the maximum range of normal strain and the range of normal stress:

(27) $$DP_{ns}={\left(\Delta \varepsilon_{n}^{}\Delta \sigma_{n}\right)}_{max}+\left(\Delta \gamma \Delta \tau_{}\right)\left(\frac{2}{1-\frac{\sigma_{n,min}}{\sigma_{n,max}}}\right).$$

The second proposal concerns the application of the product of the maximum range of the shear strain and the range of shear stresses in the form of:

(28) $$DP_{ns}=\left[{\left(\Delta \varepsilon_{n}^{}\Delta \sigma_{n}\right)}_{}+{\left(\Delta \gamma \Delta \tau_{}\right)}_{max}\right]\left(\frac{{\sigma^{\prime }}_{f}}{{\sigma^{\prime }}_{f}-{\sigma^{\prime }}_{n,mean}}\right).$$

where *σ*’*_f_* is the coefficient of the fatigue limit.

The subscript *_max_* indicates the maximum value over all planes of the quantity in the parentheses.

Chu et al. [45] proposed maximizing the sum as:

(29) $$DP_{ns}={\left(\tau_{max}\frac{\Delta \gamma_{}}{2}+\sigma_{n,max}\frac{\Delta \varepsilon_{n}}{2}\right)}_{max}$$

This parameter was successfully used in [46]. Li et al. [47] also proposed the sum of the normal and tangential components, with the tangent part being taken into account with the weighting function, Ω, as:

(30) $$DP_{n}=\frac{\Delta \varepsilon_{1}^{max}}{2}\Delta \sigma_{1}^{max}+\Omega \;\frac{\Delta \gamma_{1}^{}}{2}\frac{\Delta \tau_{1}}{2}.$$

As in [47], Pan et al. [48] introduced weighting factors, but they apply to the normal part. Finally, the damage parameter is defined as:

(31) $$DP_{ns}=\left(\frac{\Delta \sigma_{12}}{2}\frac{\Delta \gamma_{12}}{2}\right)+k_{1}k_{2}\left(\frac{\Delta \sigma_{22}}{2}\frac{\Delta \epsilon_{22}}{2}\right),$$

where the values in the critical plane are:

(32) $$k_{1}=\frac{{\gamma^{\prime }}_{f}}{{\varepsilon^{\prime }}_{f}},$$

(33) $$k_{2}=\frac{{\sigma^{\prime }}_{f}}{{\tau^{\prime }}_{f}}.$$

Rolovic and Tipton [49] proposed the general form of the damage parameter, which is presented as the sum of the strain energy density and the complementary strain energy density imposed by the stresses and strains acting on the critical plane:

(34) $$\left[\tau_{a}+f_{1}\left(\sigma_{n}\right)\right]\gamma_{a}+\left[\sigma_{n,a}+f_{2}\left(\sigma_{n}\right)\right]\varepsilon_{n,a}=f_{3}\left(N_{f}\right).$$

A specific form of Equation (34) proposed in [49] is presented as follows:

(35) $$DP_{ns}=\left[\tau_{a}+0.3\left(\sigma_{n}\right)\right]\gamma_{a}+\sigma_{n,max}\varepsilon_{n,a}.$$

Ince and Glinka [50] proposed taking into account different energy components in the form of:

(36) $$DP_{ns}={\left(\tau_{max}\frac{\Delta \gamma_{e}}{2}+\frac{\Delta \tau_{}}{2}\frac{\Delta \gamma_{p}}{2}+\sigma_{n,max}\frac{\Delta \varepsilon_{n,e}}{2}+\frac{\Delta \sigma_{n}}{2}\frac{\Delta \epsilon_{n,p}}{2}\right)}_{max}$$

looking for the maximum value in the sum, as in Chu et al.’s work—Equation (29). Jiang et al. [51,52] modified the Smith-Watson-Topper parameter to the form:

(37) $$DP_{ns}=2b\Delta \varepsilon \sigma_{max}+\frac{1-b}{2}\Delta \tau \Delta \gamma ,$$

where *b* is the material constant. The variable *b* varies from 0 to 1. For *b* = 1, the original SWT parameter is obtained. In this way, we obtain the weighting factors before both the tangential and the normal components. This equation was also successfully used in the work of Bededetti et al. [53]. Jiang and Sehitoglu [54] modified the Smith-Watson-Topper parameter to form:

(38) $$DP_{ns}=\frac{\Delta \varepsilon }{2}\langle \sigma_{max}\rangle +J\Delta \tau \Delta \gamma ,$$

where < > are MacCauley brackets:

(39) $$\langle x\rangle =0.5\left(\left|x\right|+x\right)$$

and *J* is the material constant.

This model was also successfully used by Abbadi et al. [55]. It is similar to Equation (30) with a different description of normal stresses. The model for prediction of the number of cycles to crack initiation is based on a combination between the Manson-Coffin relationship and the Jiang-Sehitoglu fatigue parameter. The Varvani-Farahani proposal [56] is also based on SWT, but the damage parameter is in the form of strain:

(40) $$DP_{\varepsilon \gamma }=\frac{1}{{\sigma^{\prime }}_{f}{\varepsilon^{\prime }}_{f}}\left(\Delta \sigma_{n}\Delta \varepsilon_{n}\right)+\frac{1+\sigma_{n}^{m}/{\sigma^{\prime }}_{f}}{{\tau^{\prime }}_{f}{\gamma^{\prime }}_{f}}\left(\Delta \tau_{max}\Delta \frac{\gamma_{max}}{2}\right),$$

where ${\sigma^{\prime }}_{f}$ is the coefficient of the fatigue limit, ${\tau^{\prime }}_{f}$ is the shear coefficient of the fatigue limit, ${\varepsilon^{\prime }}_{f}$ is the coefficient of the plastic fatigue strain, and ${\gamma^{\prime }}_{f}$ is the coefficient of the plastic shear fatigue strain.

There is also a modified version [14] formulated in the energy form:

(41) $$DP_{ns}=k_{2}\left(\Delta \sigma_{n}\Delta \varepsilon_{n}\right)+\left(1+\frac{\sigma_{n}^{m}}{{\sigma^{\prime }}_{f}}\right)\left(\Delta \tau_{max}\Delta \frac{\gamma_{max}}{2}\right),$$

where

(42) $$k_{2}=\frac{{\tau^{\prime }}_{f}{\gamma^{\prime }}_{f}}{{\sigma^{\prime }}_{f}{\varepsilon^{\prime }}_{f}}.$$

The normal and shear energies used in this parameter are divided by the tensile and shear fatigue properties, respectively. According to [56], the proposed parameter successfully correlates multiaxial fatigue lives for various in-phase and out-of-phase multiaxial fatigue straining conditions, tests in which a mean stress was applied normal to the maximum shear plane, and out-of-phase tests in which there was additional hardening. Some modification of this version (40) was presented by Jahed and Varvani [57]:

(43) $$DP_{\varepsilon \gamma }=\frac{1}{{\sigma^{\prime }}_{f}{\varepsilon^{\prime }}_{f}}\left(\Delta \sigma_{n}\Delta \varepsilon_{n}\right)+\frac{1+\sigma_{n}^{m}/{\sigma^{\prime }}_{f}}{{\tau^{\prime }}_{f}{\gamma^{\prime }}_{f}}{\left(\frac{\Delta \tau_{}}{2}\frac{\Delta \gamma_{}}{2}\right)}_{max}.$$

You et al. [16] proposed another equation for the damage parameter based on Ince and Glinka’s proposals, similarly to Chu et al. [45]. This proposal concerns the critical plane determined by the maximum shear stress:

(44) $$DP_{ns}=\tau_{max}\frac{\Delta \gamma_{}}{2}+\sigma_{n,max}\frac{\Delta \varepsilon_{n}}{2}.$$

On this basis, Zhu et al. [17] proposed a correction factor in the normal component of the critical plane in the form of:

(45) $$DP_{ns}=\tau_{max}\frac{\Delta \gamma_{}}{2}+k\sigma_{n,max}\frac{\Delta \varepsilon_{n}}{2}.$$

The proposed damage parameter differentiates the effects of shear strain and normal strain on fatigue damage on the critical plane near the maximum shear strain plane. This is consistent with the proposal presented in the Equation (31).

Arora et al. [11] formulated the damage parameter using a formula analogous to Equation (37):

(46) $$DP_{ns}=\left(k-1\right)\tau_{max}\frac{\Delta \gamma_{}}{2}+k\sigma_{n,max}\frac{\Delta \varepsilon_{n}}{2}.$$

Another proposal was presented in [58] in the form of:

(47) $$DP_{ns}=\left(\tau_{max}+\frac{\sigma_{n,max}^{*2}{\tau^{\prime }}_{f}}{\sqrt{3}{\sigma^{\prime }}_{f}}\right)\sqrt{3\varepsilon_{n}^{*2}+{\left(\frac{\Delta \gamma_{max}}{2}\right)}^{2}},$$

where $\sigma_{n}^{*}$ is the normal strain excursion between adjacent turning points of the maximum shear strain range on the critical plane. The next proposal is the equation presented by Li et al. [47] in the form of:

(48) $$DP_{n}=\sqrt{{\left(\frac{\Delta \varepsilon_{1}^{max}}{2}\Delta \sigma_{1}^{max}\right)}^{2}+{\left(\frac{\Delta \gamma_{1}^{}}{2}\frac{\Delta \tau_{1}}{2}\right)}^{2}}.$$

This equation defines the damage parameter as the geometric mean of the normal and tangential components. It is found that the SWT model usually overestimates the fatigue lives of materials since it only takes into account the fatigue damage caused by the tensile components.

In [59], the authors proposed using the strain energy density parameter for the evaluation of fatigue life in a uniaxial random load state based on the product of the stress and strain history. On this basis, the multiaxial random fatigue criteria [60] based on this parameter were proposed. For cyclic loading, the above criterion is reduced to two forms, depending on the choice of the position of the critical plane. If the critical plane is defined by a normal component, the damage parameter takes the form:

(49) $$DP_{n}=\beta W_{nsa}+W_{na}$$

where *β* is the coefficient taking into account the best fit for a given material, i.e., with the smallest scatter. This coefficient is best determined from tests with phase shift π/2 and when the amplitude of the normal tangential component is given by the equations:

(50) $$W_{nsa}=\sigma_{nsa}\gamma_{nsa}\;$$

(51) $$W_{na}=\sigma_{na}\varepsilon_{na}\;\;\;$$

However, if the critical plane is defined by the amplitude of the tangential component, the damage parameter is given by the equation:

(52) $$DP_{n}=\beta W_{nsa}+\frac{4-\beta \left(1+\vartheta \right)}{1+\vartheta }W_{na},$$

where

(53) $$\beta =\frac{\sigma_{af}^{2}}{\tau_{af}^{2}\left(1+\vartheta \right)}.$$

In [61], applying the damage parameters (49) and (52) in a complex loading state for a range of small number of cycles was proposed. In the case of the critical plane defined by the normal component, the form (49) is not modified. However, if the critical plane is defined by the tangential component, the damage parameter (52) can be formulated as:

(54) $$DP_{n}=2\frac{\sigma_{af}\varepsilon_{af}}{\tau_{af}\gamma_{af}}W_{nsa}+\frac{4}{1-\gamma_{eff}}\left[1-\frac{\left(1+\vartheta_{eff}\right)\sigma_{af}\varepsilon_{af}}{2\tau_{af}\gamma_{af}}\right]W_{na}.$$

## 6. Energy Fatigue Characteristics

In earlier models, the damage parameters determined (stress–strain) are, in effect, respectively defined as normal equivalents, tangential equivalents, or tangential and normal equivalents. Therefore, they must be compared with the corresponding fatigue characteristics. As a result, if amplitudes or maximum values are used in setting the parameters, the fatigue characteristics are based on the classic Manson-Coffin-Basquin characteristic:

(55) $$\varepsilon_{a}=\varepsilon_{a,e}+\varepsilon_{a,p}=\frac{{\sigma^{\prime }}_{f}}{E}{\left(2N_{f}\right)}^{b}+{\varepsilon^{\prime }}_{f}{\left(2N_{f}\right)}^{c}$$

where *b* is the exponent of the fatigue limit, and *c* is the exponent of the plastic fatigue strain.

The fatigue characteristics can also be based on the Basquin equation in tension:

(56) $$DP_{\sigma }={\sigma^{\prime }}_{f}{\left(2N_{f}\right)}^{b}$$

An analogous characteristic to Equation (55) can be formulated in the shear strain notation as follows:

(57) $$\gamma_{a}=\gamma_{a,e}+\gamma_{a,p}=\frac{{\tau^{\prime }}_{f}}{G}{\left(2N_{f}\right)}^{b_{o}}+{\gamma^{\prime }}_{f}{\left(2N_{f}\right)}^{c_{o}}$$

where *b_o_* is the exponent of the shear fatigue limit, and *c_o_* is the exponent of the plastic shear fatigue strain.

They can also be defined in the shear stress notation analogous to Equation (56):

(58) $$DP_{\tau }={\tau^{\prime }}_{f}{\left(2N_{f}\right)}^{b_{o}}.$$

The damage parameters shown above relate to normal and shear strains (Equations (55) and (57)) and normal and shear stresses (Equations (56) and (58)). On the basis of Equations (55) and (56), it is possible to determine the damage parameter according to the SWT parameter defined in the normal component:

(59) $$DP_{n}=\frac{{\sigma^{\prime }}_{f}^{2}}{E}{\left(2N_{f}\right)}^{2b}+{\sigma^{\prime }}_{f}{\varepsilon^{\prime }}_{f}{\left(2N_{f}\right)}^{b+c}.$$

or pursuant to Equations (56) and (57) on the basis of the parameter defined in the tangential component, we obtain the shear damage parameter:

(60) $$DP_{s}=\frac{{\tau^{\prime }}_{f}^{2}}{G}{\left(2N_{f}\right)}^{2b_{o}}+\frac{{\tau^{\prime }}_{f}{\gamma^{\prime }}_{f}}{2}{\left(2N_{f}\right)}^{b_{o}+c_{o}}$$

The strain parameter based on normal (55) and shear (57) strains may be defined as:

(61) $$DP_{\varepsilon \gamma }=\frac{{\sigma^{\prime }}_{f}}{E}{\left(2N_{f}\right)}^{b}+{\varepsilon^{\prime }}_{f}{\left(2N_{f}\right)}^{c}+\frac{{\tau^{\prime }}_{f}}{G}{\left(2N_{f}\right)}^{b_{o}}+{\gamma^{\prime }}_{f}{\left(2N_{f}\right)}^{c_{o}}$$

and a combination of parameters based on the normal (59) and tangential (60) components:

(62) $$DP_{n,s}=\frac{{\sigma^{\prime }}_{f}^{2}}{E}{\left(2N_{f}\right)}^{2b}+{\sigma^{\prime }}_{f}{\varepsilon^{\prime }}_{f}{\left(2N_{f}\right)}^{b+c}+\frac{{\tau^{\prime }}_{f}^{2}}{G}{\left(2N_{f}\right)}^{2b_{o}}+\frac{{\tau^{\prime }}_{f}{\gamma^{\prime }}_{f}}{2}{\left(2N_{f}\right)}^{b_{o}+c_{o}}$$

As can be seen, all these characteristics have a non-linear form due to the number of cycles. In some works (e.g., [62,63,64]), it was noted that in some sections it can be determined as a linear function in a double logarithmic system in a general power form, as:

(63) $$DP=A{\left(N_{f}\right)}^{B}.$$

This characteristic has no physical significance and is purely empirical, but can be very convenient in calculations, and in particular can be effective in analytical calculations. Its efficiency can be very useful in the analysis of materials in an elastic state.

## 7. Discussion

Since the problem of the degradation of materials under cyclical loading was first identified, a significant number of models for estimating fatigue life have been proposed, but none of them can be widely accepted as universal. Authors around the world are making efforts to modify and extend existing theories to include all the variables that play a key role in cyclic load changes. The complexity of the material fatigue problem makes this topic current, and interesting theories using new approaches are constantly emerging. The scope of application of each model varies from case to case and depends on the specific application and factors to be taken into account. One of the most frequently modified fatigue life estimation models is the Smith-Watson-Topper model (SWT). The SWT parameter in its original form was dedicated to estimating the fatigue life of metal materials in a uniaxial load state (tension–compression) in the range up to fatigue crack initiation, with non-zero mean values. It seems that the original version of this parameter (also with modifications only in the normal direction) can only be used in the case of a uniaxial load condition. Then, Equation (56) or (58) can be used to estimate the fatigue life if the body of the elastic model is adopted. Then, Equation (56) can be applied if the damage parameter was previously determined as normal stress by Equations (5), (7), (10)–(13), or (58) is determined as shear stress (8, 15). Similarly, when adopting the model of an elastic-plastic body, the fatigue life can be determined according to Equation (59) if the energy parameter in the normal direction, defined, for example, according to Equations (4), (30), (48), (49), (52), (54), or Equation (60), was chosen as the damage parameter, with the energy parameter defined by Equations (14) and (17)–(19). Future modifications extended the applicability of the model to other types of loads such as torsion and multiaxial loads. With the development of knowledge about fatigue failure, models based on the critical plane theory, which is characterized by the assumption that the stress state components associated with the plane of a certain orientation are responsible for the initiation of fatigue cracking, became more and more popular. Researchers further developed the SWT parameter by making the material strength limits dependent on defining their occurrence in the normal or shear plane. Subsequent modifications concerned the influence of non-zero values of mean loads. In these modifications, additional static values were taken into account in whole or in part; however, partial consideration of mean stresses was very often carried out not mathematically but empirically, by the best matching of the reducing factor to the experimental results. Many researchers succeeded in implementing successive modifications of the SWT parameter for multiaxial loads, in which the static stresses were taken into account in their entirety or reduced by the mean effect factor. As interest in the industry increased for materials other than homogeneous metals, subsequent modifications were performed in an attempt to implement Smith, Watson, and Topper’s idea for composite materials. These modifications required significant changes due to the directionality of material properties being significantly different from homogeneous materials. Analyzing all of the damage parameters and the corresponding fatigue characteristics, it seems that the parameters in which the fatigue life is determined from the fatigue characteristics that arise on the basis of two load states (tension–compression and double-sided shearing) have the greatest potential. This phenomenon must be addressed, regardless of whether the damage parameter is defined by the strain parameter (61) or the energy parameter (62). The deformation model (61) is proposed relatively rare—Equations (40) and (43). On the other hand, the energy model (62) is the most commonly used—Equations (24)–(29), (31), (36)–(38), (41), (44)–(47). In future calculations of the available results, the focus should be on the verification and development of this model.

## 8. Conclusions

After having analyzed the presented fatigue life estimation models, the following conclusions can be drawn:

- By studying the literature on fatigue failure of materials, one can notice a significant increase in the number of fatigue life estimation models, which at first glance seem to be new ideas, but analyzing them carefully, one can see that some of them are based on the same idea as that proposed by Smith-Watson-Topper (SWT), which has been presented in detail in this paper.
- All proposals related to the analyzed SWT parameter were defined in the critical plane.
- By analyzing all the damage parameters and the corresponding fatigue characteristics, it can be concluded that parameters in which the fatigue life is determined from the fatigue characteristics that arise on the basis of two load states (tension–compression and bilateral shear) have the greatest potential.
- In the future, calculations should be performed according to the selected presented models, based on the available experimental research, with particular emphasis on two fatigue characteristics.

## Data Availability

Not applicable.

## Nomenclature

*b*	exponent of the fatigue limit
*b_o_*	exponent of the shear fatigue limit
*c*	exponent of the plastic fatigue strain
*c_o_*	exponent of the plastic shear fatigue strain
*DP_n_*	damage parameter
*E*	Young’s modulus of elasticity
*G*	shear modulus
*J*	material constant
*k*	material fitting parameter
*N_f_*	number of cycles to failure
*β*	coefficient taking into account the best fit for a given material
$d\varepsilon_{ij}^{p}$	plastic strain tensor
$\varepsilon_{a}$	normal strain amplitude
*ε_n_**	normal strain excursion between adjacent turning points of the maximum shear strain range on the critical plane
${\varepsilon^{\prime }}_{f}$	coefficient of the plastic fatigue strain
γ	material-dependent exponent
$\gamma_{a}$	shear strain amplitude
${\gamma^{\prime }}_{f}$	coefficient of the plastic shear fatigue strain
$\gamma_{max}$	maximum shear strain
${\sigma^{\prime }}_{f}$	coefficient of the fatigue limit
$\sigma_{ij}$	stress tensor
*σ_h_*	hydrostatic stress
$\sigma_{max}$	maximum normal stress
*σ_u_*	ultimate strength
*σ_y_*	yield strength
(*στ*)*_max_*	maximum shear and tensile stress product value
${\tau^{\prime }}_{f}$	shear coefficient of the fatigue limit
$\tau_{max}$	maximum shear stress
*υ*	elastic Poisson’s ratio
